# Revenge or collusion? An experiment on payoff subtraction and addition in team contests

**DOI:** 10.1371/journal.pone.0331015

**Published:** 2025-09-04

**Authors:** Jiaxin Yu, Wanjun Zheng

**Affiliations:** 1 Business School, Yangzhou University, Yangzhou, China; 2 Center for Economic Behavior and Decision-Making, Zhejiang University of Finance and Economics, Hangzhou, China; 3 School of Economics, Zhejiang University of Finance and Economics, Hangzhou, China; Universitat Jaume I, SPAIN

## Abstract

Group competition is prevalent in contemporary society. In this paper, we focus on the effects of payoff subtraction (revenge) and payoff addition (collusion) on expenditures in team competitions. Although theory predicts that the equilibrium of aggregate team contributions does not change, we find that competitive expenditures are indeed affected. Our results indicate that (1) embedding the payoff subtractions that target either the top investor or the entire team significantly reduces the contest expenditures of the targeted group; (2) targeting the top investor with conditional payoff subtractions (depending on relative expenses) leads to a slight reduction in total expenditures across both competing parties; and (3) if the primary objective is to reduce competitive expenditures, either to lower the rival group’s expenditures or to lower that of both sides, payoff additions of equal magnitude may be less effective than payoff subtractions. These findings provide valuable insights into potential mechanisms for reducing competitive resource waste.

## 1. Introduction

Competition is prevalent in contemporary society, with countries, political parties, trade unions, and even employees often engaging in forms of it. Research has shown that competition between groups that rely exclusively on the voluntary contribution of their members to the collective effort is characterized by a much higher than equilibrium level of commitment to fighting, which leads to a significant waste of resources [1–4].

Thus, it is imperative to examine the effective mechanisms of resolving conflicts between groups to avoid wasting resources. The significant over-expenditure of effort in contests can be explained in three ways, namely, overly competitive behaviour, which is usually observed when individuals compete against others in contests; overly cooperative behaviour, which is usually observed in social dilemmas and collective action games; and parochial altruism and social identity [2,4]. Some studies have proposed several effective ways to resolve conflicts between individuals, including communication between competing parties [5,6], side payments [7,8], the use of a coordination device [9], and costly commitments [10]. Unfortunately, conflict resolution mechanisms are rarely studied in the context of group contests. Cason et al. [6] reported that communication between groups can de-escalate and potentially eliminate between-group conflict. As intergroup conflict is an important form of social conflict, it is still an area open to future research.

To bridge this gap, this study aims to explore potential mechanisms to reduce expenditures, particularly in group contests. It is well established that sanctions are effective ways to sustain cooperation [11–13]. The presence of either positive (i.e., rewards or carrots) or negative (i.e., punishments or sticks) sanctions has been shown to influence cooperative behavior in public good problems. Additionally, Dari-Mattiacci and Geest [14] highlighted the “multiplication effect” of negative sanctions, suggesting that the mere possibility of punishment is often sufficient to enforce cooperation, even if the sanction itself is rarely applied. Costly sanctions, such as within-group punishments, have also been shown to escalate competition, although little research has been conducted. Abbink et al. [1] found that when group members could punish each other, competitive expenditures more than doubled compared with those in settings without punishment, resulting in substantial resource dissipation. In addition, noncostly sanctions, such as social disapproval, could influence team competition. Sutter et al. [15] showed that communication between competing teams may lead to collusion, thereby reducing competitive expenditures.

Building on this theoretical foundation, this study applies positive and negative sanctions to competitive environments, adapting them as mechanisms to reduce competitive expenditures. Specifically, we examine the effects of payoff subtraction (revenge) and payoff addition (collusion) mechanisms on individual and team-level competitive expenditures. Payoff subtraction (revenge) refers to intergroup revenge, in which competing groups impose penalties on those who have made higher expenditures. This mechanism may serve as a deterrent, discouraging excessive expenditure by introducing the risk of payoff subtractions. Additionally, the payoff subtraction treatments can be further classified into conditional and unconditional payoff subtractions. Unconditional revenge applies penalties indiscriminately, regardless of the expenditure level. Conditional revenge is triggered only when expenditures exceed a specific threshold, making it a more strategic and targeted response.

The payoff addition (collusion), in contrast, operates through payoff addition rather than subtraction. Instead of punishing high investors, it incentivizes lower expenditures by granting selective rewards to those who invest less. This may create an alternative pathway to reduce competition, as players may strategically adjust their expenditures in anticipation of receiving these benefits. Like revenge, collusion can also be conditional or unconditional, depending on whether the reward is granted automatically or contingent on specific expenditure conditions. Both revenge and collusion fundamentally alter team incentives, potentially disrupting excessive cooperation within groups and reshaping competitive dynamics.

The different treatments in our experiment were designed to reflect a range of real-world strategic interventions commonly observed in intergroup conflicts. The standard competition is analogous to unfettered rivalry in markets or politics. The revenge treatments simulate punitive strategies, in which unconditional revenge mimics broad sanctions or deterrence measures, and conditional revenge mirrors targeted response triggered by the opponent’s escalation, as seen in diplomatic or military standoffs. The collusion treatments represent cooperative incentives. The unconditional collusion rewards restraint regardless of group behavior, whereas the conditional collusion ties support to the collective behavior of the rival group, reflecting contingent peace-building or diplomatic aid strategies. By capturing these varied approaches, our design allows for a systematic comparison of how different payoff structures shape competitive behavior and resource expenditure.

Our paper contributes to the literature concerning costly sanctions in group contests. Prior research has examined the role of both costly and non-costly sanctions in shaping behaviour within competition environments [1,15]. In group contests, competitive pressures and strategic incentives often drive participants to overinvest in conflict, leading to excessive expenditures. While previous studies have shown that punishment can sometimes exacerbate wasteful investments, our study takes a different approach by introducing targeted payoff interventions. We investigated whether strategic payoff subtractions (revenge) or additions (collusion) can influence competitive behaviour and potentially reduce resource over-expenditures. Our findings provide new insights into how costly sanctions shape contest dynamics, offering potential mechanisms to reduce resource dissipation in group competition.

The rest of the paper is organized as follows: Section 2 describes the methods, including the experimental design, model and procedure used. Section 3 reports the results of the experiment. Section 4 offers alternative explanations for the intervention on over-expenditure observed in the experiment.

## 2 Method

### 2.1 Subjects

A total of 360 college students (136 females; mean age 20.6 years, ranging from 18 to 29 years) participated in our experiment. Participants were recruited from a university-wide subject pool from 18 March to 15 April 2024. The experiments lasted approximately 60 min, and the average payment was 58.8 RMB yuan (approximately 8.1 US dollars), which fluctuated according to performance. The participants provided informed written consent before the experiment. The experiment was approved by the Zhejiang University of Finance and Economics ethics committee (2024031604).

### 2.2 Experimental design

#### 2.2.1 General framework.

The experiment was adapted from a Tullock contest game [16]. The contest game was held between two parties, namely, Team X and Team Y. Team X consisted of $N_{X}$ individuals, whereas Team Y had $N_{Y}$ individuals.

At the beginning of each round, the initial endowment of both teams was the same, denoted *E*. The participants on Team X each received an endowment of $E/N_{X}$ points, whereas the participants on Team Y received an endowment of $E/N_{Y}$ points. Each participant could use their points to buy contest tokens for their parties. After everyone had decided, the computer determined the winning team. The members of the winning party received an extra *P* points each, regardless of the team size. The probability of winning was related to the contest tokens of both teams, which was calculated as follows. The probability of Team X obtaining the prize was X/(X+Y). The probability of Team Y winning the prize was Y/(X+Y). For example, if Team X invested a total of 150 tokens and Team Y invested 100 tokens, then the probability of Team X receiving the prize would be 3/5 and the probability of Team Y receiving the prize would be 2/5. If Team X and Team Y invested the same number of tokens, that is, X=Y, then Team X and Team Y would have the same probability of winning the prize, i.e., 1/2. However, if the expenditures of both teams were 0, then neither would receive the prize.

#### 2.2.2 Equilibrium analysis for the general framework.

We first consider the general framework. Let $\pi_{i}\left(x_{i},X,Y\right)$ denote the payoff of a representative player i on Team X, where $x_{i}$ is the number of contest tokens purchased by player i. E is the initial endowment, and P is the winning prize. X is the sum of contest tokens purchased by player i ’s team, and Y is the total number of contest tokens bought by the opponent party. The payoff function of player i on Team X can then be written as follows:


$$\pi_{i}\left(x_{i},X,Y\right)=\frac{E}{N_{X}}+\frac{X}{X+Y}\times P-x_{i}.$$

Let $\pi_{j}\left(y_{j},X,Y\right)$ denote the payoff of player j on Team Y. The player’s payoff function in this game can then be written as follows:


$$\pi_{j}\left(y_{j},X,Y\right)=\frac{E}{N_{Y}}+\frac{Y}{X+Y}\times P-y_{j}.$$

The first-order condition for a representative member of Team X, derived in the usual way, is $PY={\left(X+Y\right)}^{2}$. The same calculation is performed for members of Team Y, yielding $PX={\left(X+Y\right)}^{2}$. Therefore, we have $X^{*}=Y^{*}=0.25P$ as the equilibrium prediction. Note that the equilibrium contains only aggregate team contributions. In fact, this game has multiple equilibria; as long as the total contributions of Team X sum to 0.25P, then it is an equilibrium. Additionally, the equilibrium is independent of the team size. At equilibrium, the aggregate team contributions are the same whether the team has four members or only one member.

In our experiment, Team X consisted of four individuals, whereas Team Y had only one individual. The endowment for both teams was set to 200; therefore, participants on Team X each received an endowment of 50 points, and the only participant on Team Y received an endowment of 200 points. The prize (P) for the winning team received was extra 100 points each. The group without any payoff addition or subtraction serves as the benchmark and is referred to as the baseline treatment. In the **baseline treatment**, following the equilibrium analysis for the general framework, the first-order conditions for a representative member of Team X and the participant on Team Y are $PY={\left(X+Y\right)}^{2}$ and $PX={\left(X+Y\right)}^{2}$, respectively. Therefore, we have $X^{*}=Y^{*}=\frac{1}{4}P=25$ as the equilibrium prediction.

#### 2.2.3 Corner solution in the general framework.

Now, let us consider the individual behaviors of Team X. Their contributions at equilibrium can be written as


$${x_{i}}^{*}=\frac{1}{4}P-\left(n-1\right)\bar{x},$$

where $\bar{x}$ is the mean contribution of all other teammates. We can see that when the sum of others’ contributions is equal to or greater than $\frac{1}{4}P$, the equilibrium of player i ’s contribution is 0, which means a rational exit for player i.

Note that in the Nash case, individuals behave in a naïve fashion: they conjecture that a change in their $x_{i}$ does not affect $\bar{x}$ (the behaviour of others). We can also relax the zero conjectural variation as [17], which in fact may be more consistent with our repeated games. Let $\beta_{i}$ be player i ’s conjectural variation with respect to the mean activity of other teammates, i.e., $\beta_{i}=\frac{\partial \bar{x}}{\partial x_{i}}$. At equilibrium, we have $X^{*}=\left[1+\left(n-1\right)\beta_{i}\right]Y^{*}$ (see S1 Appendix for details). That means an exit for everyone in Team X when $\beta_{i}\leq -1/\left(n-1\right)$, and at least one member entries when $\beta_{i}>-1/\left(n-1\right)$. Clearly, if $\beta_{i}=0$, we have the Nash equilibrium that $X^{*}=Y^{*}=1/4P$. At the individual level, we have (see S1 Appendix for details). If the sum of others’ contributions is equal to or greater than $\frac{1}{1+\frac{2}{1+\left(n-1\right)\beta_{i}}+\frac{1}{{\left(1+\left(n-1\right)\beta_{i}\right)}^{2}}}P$, then the equilibrium of player i ’s contribution would be 0.

#### 2.2.4 Experimental treatments and equilibrium analysis.

Our design was composed of nine treatments, namely, one baseline treatment, four treatments with revenge, and four treatments with collusion. The participants were randomly assigned to one of these treatments. They interacted with fixed parties and with a fixed opponent party during the 20 rounds of the experiment.

**Fig 1:**
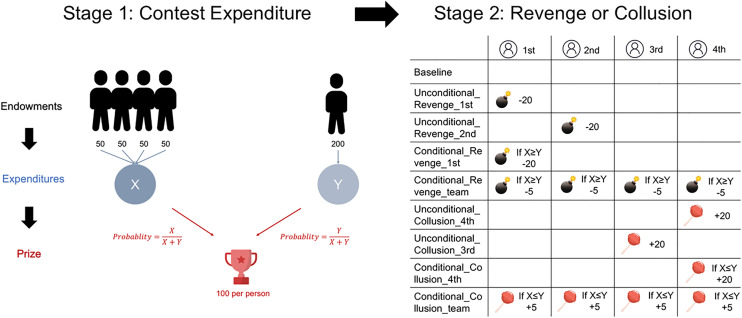
Timeline of the experiment. Each trial contains two stages. Stage 1 represents the contest where Team X and Team Y compete for the prize. Stage 2 introduces different treatments: Revenge (pay-off subtractions) and Collusion (pay-off additions), where decisions can be either conditional (based on relative expenditures) or unconditional.

The participants must decide how much of their endowment to put aside as competition expenses, which influences the probability of winning the prize. The participants who were assigned to the revenge or collusion treatments moved to the revenge or collusion stage after completing the contest expenditure stage in each round. Fig 1 shows the experimental timeline.

We designed four different revenge treatments, two for unconditional revenge and two for conditional revenge. In the first unconditional revenge (**Unconditional_Revenge_1st**), the member of Team X who had invested the greatest number of tokens in the competition stage experienced revenge against Team Y, resulting in a loss of 20 tokens. If n individuals (n ≥ 2) invested equally and the highest out of the group, then all of these people would have a probability of 1/n of experiencing revenge. In the first unconditional revenge (**Unconditional_Revenge_2nd**), the member of Team X who had invested the second highest number of tokens in the competition stage suffered revenge, resulting in a loss of 20 tokens. If n individuals (n ≥ 2) invested equally and second most in the group, then all of these people would have a probability of 1/n of experiencing revenge.

Conditional revenge means that revenge will occur only if conditions are triggered. In the first unconditional revenge (**Conditional_Revenge_1st**), if the total number of tokens invested by Team X was not lower than that invested by Team Y, then the member who invested the most out of Team X would suffer a loss of 20 tokens. If n individuals (n ≥ 2) invested equally and the most out of the group, then all of these people would have a probability of 1/n of experiencing revenge. Conversely, the revenge would be cancelled. In the second unconditional revenge (**Conditional_Revenge_Team**), if the total number of tokens invested by Team X was not lower than that invested by Team Y, then all the members of Team X would experience revenge, with a loss of 5 tokens each. Conversely, the revenge would be cancelled.

Then we considered equilibrium analysis for revenge treatments. In **Unconditional_Revenge_1st**, we assume that the contributions of the four players on Team X satisfy $x_{1}>x_{2}>x_{3}>x_{4}$. Then, the payoff function of the player who invested most on Team X can be written as follows:


$$\pi_{1}\left(x_{1},X,Y\right)=50+\frac{X}{X+Y}\times 100-x_{1}-20.$$

The payoff function of the other players (i=2,3,4) on Team X can be written as follows:


$$\pi_{i}\left(x_{i},X,Y\right)=50+\frac{X}{X+Y}\times 100-x_{i}.$$

The only player’s payoff function on Team Y can be written as follows:


$$\pi_{Y}\left(X,Y\right)=200+\frac{Y}{X+Y}\times 100-Y$$

Under Nash equilibrium, the first-order conditions result in an interior equilibrium where $X^{*}=Y^{*}=25$. The Nash equilibrium does not change when more than one person puts in not only an equal amount but also the largest amount of tokens from Team X. A similar calculation can be made for the other three revenge rules, and we must have $X^{*}=Y^{*}=25$ in Nash equilibrium. When we allow for general conjectures, the equilibrium for the sum of team X would be higher than 25, and Team X invests more than Team Y when $\beta_{i}>0$; and the equilibrium for the sum of team X would be lower than 25, and Team X invests less than Team Y when $\beta_{i}<0$ (See S1 Appendix for details). Note that the equilibrium prediction is not affected by the prospect of a pay-off deduction.

Similarly, we also designed four different collusion treatments, two for unconditional collusion and two for conditional collusion. In the first unconditional collusion (**Unconditional_Collusion_4th**), the member who invested the least number of tokens in the previous competition stage would be solicited for an additional 20 tokens. If n individuals (n ≥ 2) invested equally and the least out of the group, then each person would have a probability of 1/n of being solicited. In the second unconditional collusion (**Unconditional_Collusion_3rd**), 20 tokens were added to the member of Team X, who had invested the second lowest number of tokens in the competition stage. If n individuals (n ≥ 2) invested equally and the second least out of the group, then each person would have a probability of 1/n of being solicited.

In the first unconditional collusion (**Conditional_Collusion_4th**), if the total number of tokens invested by Team X was not higher than that of Team Y, then the member who invested the least on Team X would be solicited for an additional 20 tokens. If n individuals (n ≥ 2) invested equally and the least out of the group, then each person had a probability of 1/n of being solicited. Conversely, the collusion would be cancelled. In the other unconditional collusion (**Conditional_Collusion_Team**), if the total number of tokens invested by Team X was not higher than that of Team Y, then all members of Team X would be drawn in and solicited for 5 tokens each. Conversely, the collusion would be cancelled. The outcomes of the pay-off subtractions or additions from the revenge or collusion stages are summarized Stage 2 in Fig 1.

Similar equilibrium analysis was conducted for collusion treatments. In **Unconditional_Collusion_1st**, we assume that the contributions of the four players on Team X satisfy $x_{1}>x_{2}>x_{3}>x_{4}$. The payoff function of the player who invested least on team X can be written as follows:


$$\pi_{4}\left(x_{4},X,Y\right)=50+\frac{X}{X+Y}\times 100-x_{4}+20.$$

The payoff function of the other players (i=1,2,3) on Team X can be written as follows:


$$\pi_{i}\left(x_{i},X,Y\right)=50+\frac{X}{X+Y}\times 100-x_{i}.$$

The only player’s payoff function on Team Y can be written as follows:


$$\pi_{Y}\left(X,Y\right)=200+\frac{Y}{X+Y}\times 100-Y$$

For Nash equilibrium, the first-order conditions result in an interior equilibrium where $X^{*}=Y^{*}=25$. The Nash equilibrium does not change when more than one person puts in not only an equal amount but also the least amount of tokens from Team X. A similar calculation can be made for the other three collusion rules, and we must have $X^{*}=Y^{*}=25$ in Nash equilibrium. The equilibrium prediction is also not affected by the prospect of pay-off additions.

The key equilibrium predictions of the models are as follows:

(1) As long as both groups have sufficient endowments and each individual faces the same incentives, the equilibrium contributions are the same for both teams and are unaffected by the team size.
(2) As long as both groups have sufficient endowments, the equilibrium prediction is not affected by the existence of intergroup revenge.
(3) As long as both groups have sufficient endowments, the equilibrium prediction is not affected by the existence of intergroup collusion.

The research questions emerge directly from the abovementioned equilibrium analysis

**Question 1**: Will the team party invest more or less than the individual party?

The Nash equilibrium suggests equal contributions of both teams. However, our extended model allows for interdependence within the team. In particular, if individuals expect that increasing their own effort will positively influence the average effort of others (i.e., they hold a positive conjectural variation), then contributing more becomes strategically advantageous, which potentially leads to higher overall team investment. As a result, the relative level of investment between the team and individual parties is no longer determined solely by structural incentives, but also by behavioral expectations within the team.

**Question 2**: How do payoff subtractions affect individuals’ expenditures?

Although the equilibrium of aggregate team contributions remains unchanged, individual decision-making is known to be influenced by competing incentives. On the one hand, individuals are motivated to increase their expenditures to maximize their chances of winning the prize. On the other hand, the presence of payoff subtractions (revenge mechanisms) creates a disincentive for high contributions, as individuals may seek to avoid becoming the target of deductions. The trade-off may influence individual and team-level expenditures.

**Question 3**: How do payoff additions affect individuals’ expenditures?

The impact of payoff additions on individual behaviors should not be overlooked. Experimental studies on public goods game suggest that positive sanctions (rewards) are typically used as selective incentives to influence cooperative behaviors. The selective nature of collusion, where certain individuals or the entire team receive additional payoffs, may weaken internal cooperation and reduce expenditures. However, some studies have shown that sticks have a greater effect on cooperative behavior than carrots do. Therefore, it is interesting to know whether there is asymmetry between payoff addition and payoff subtraction.

### 2.3 Procedures

All the participants were randomly assigned to one of the nine treatments. For each treatment, all groups and matches were randomly assigned and held fixed throughout the 20 rounds of the experiment. On arrival, the subjects were seated at visually separated computer terminals, and communication between them was prohibited.

All the experiments were computerized via z-Tree software [18]. The experiment began with a brief introduction to the instructions. The experiment officially started only after all the subjects fully understood it. At the beginning, each subject was informed of his or her group (Team X or Team Y) and initial endowment, which was displayed on the computer. In each round, the subjects had to choose the number of competition tokens they decided to invest. As soon as every participant had made their decision, each participant was informed about the number of competition tokens invested by their own group and by the competing group, their group’s probability of winning, the outcome of the competition and their individual earnings. In particular, the expenditures of each member of Team X were ranked by the amount of expenditures, so that others’ behaviour could be traced over time. The participants in the revenge and collusion treatments were also told about the outcomes of extra losses or gains in the revenge or collusion stage. At the end of the experiment, the subjects were informed of the total number of tokens they had made during the experiment.

## 3. Results

### 3.1 Summary of treatment effects

Before analyzing specific behavioral mechanisms in the payoff subtraction (revenge) and payoff addition (collusion) treatments, we begin by summarizing the average contribution behaviors across all treatment conditions. In the baseline treatment, where players interacted without any form of payoff subtraction or addition, average contributions were the highest: 56.50 for Team X and 50.84 for Team Y. Under the revenge treatments, where contributions could reduce the payoffs of members in Team X, average contributions declined to 43.81 for Team X and 46.84 for Team Y. In the collusion treatments, which allowed for payoff addition, the average contributions were slightly lower: 47.49 for Team X and 44.06 for Team Y. It suggested that both forms of payoff manipulation substantially reduced cooperative behavior.

**Fig 2:**
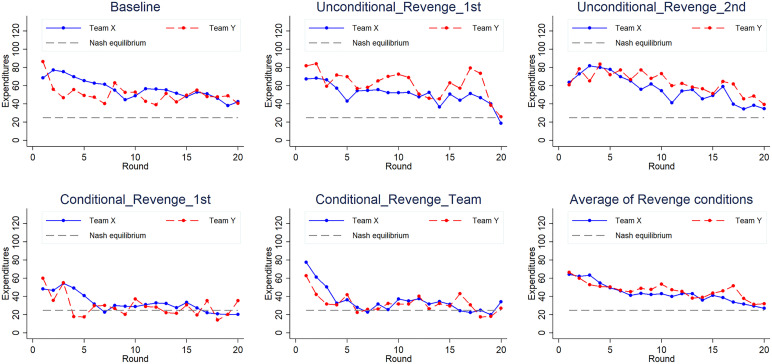
Expenditures over time in the baseline and payoff subtraction treatments. The y-axis represents expenditures, while the x-axis denotes the round number. The blue solid line depicts the total expenditure of Team X, the red dotted line represents the total expenditure of Team Y, and the grey dotted line indicates the Nash equilibrium level.

The average contribution patterns of Team X and Team Y across 20 rounds in the revenge conditions revealed a steadily downwards trend over time (see the final panel of Fig 2). Although the dynamics vary, both teams’ expenditures gradually approach the Nash equilibrium level, yet remain consistently above it throughout the game. A similar pattern emerges under collusion conditions (see the final panel of Fig 4). However, under revenge conditions, Team Y invested more than Team X on more occasions, while under collusion conditions, the opposite was true.

**Fig 3:**
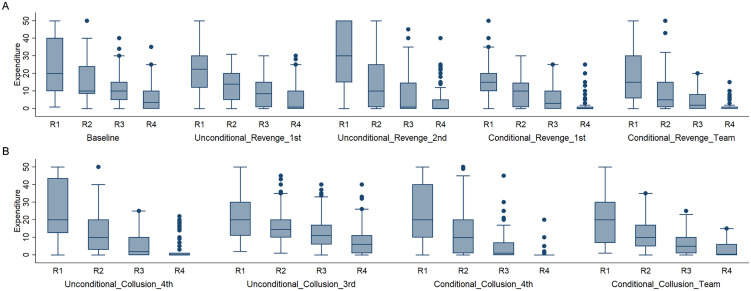
Average expenditures of ranked team players in Team X across different treatments. Panel (A) shows average expenditures of ranked team players in the baseline and payoff subtraction (Revenge) treatments, while panel (B) presents expenditures in the payoff addition (collusion) treatments. The y-axis represents expenditure levels, and the x-axis denotes ranks within the team, where R1 corresponds to the highest-ranked player in the team and R4 to the lowest-ranked player. Box plots display the distribution of expenditures, with the central line indicating the median, the box representing the interquartile range, and dots showing outliers.

**Fig 4:**
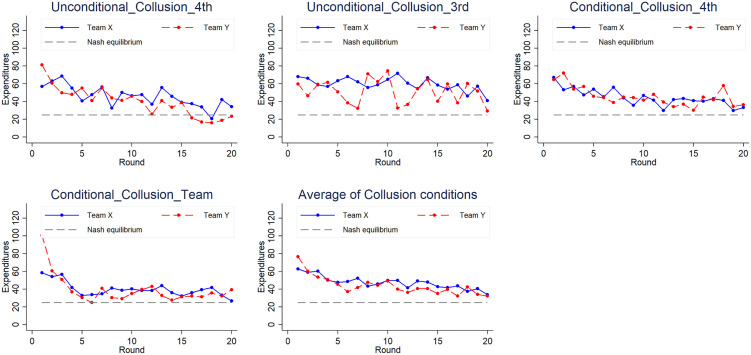
Expenditures over time in the payoff addition treatments. The y-axis represents expenditures, while the x-axis denotes the round number. The blue solid line depicts the total expenditure of Team X, the red dotted line represents the total expenditure of Team Y, and the gray dotted line indicates the Nash equilibrium level.

The remainder of Section 3 provides a more detailed breakdown of behavioral patterns under each treatment. Section 3.2 examines how revenge mechanisms influence behavior over time, while Section 3.3 focuses on the dynamics under collusion.

### 3.2 Effects of revenge

#### 3.2.1 Time trends and differences between treatments and between conflict parties.

On the basis of fig 2, which shows how the average competition token expenditures of the two competing groups evolved over time in the baseline treatment and the four revenge treatments, three observations seem noteworthy. First, there is a downwards trend over time in all the treatments. Second, the overall actual expenditures appear to be higher than the Nash equilibrium prediction. Note that since the expenditures were used for appropriation of the prize and not its production, a higher expenditure level implies a lower efficiency level. Even towards the end of the experiment, the expenditures in the baseline treatment, and the two unconditional revenge treatments appear to be greater than those in the equilibrium prediction. The expenditures seem closer to the equilibrium prediction in the two conditional revenge treatments than in the other treatments, especially in the second half of the rounds. Third, Team X seems to invest more than Team Y in the baseline treatment; however, the opposite is true in the two unconditional revenge treatments. Next, we attempt to perform a statistical analysis to determine the underlying regularity.

First, we compare differences in expenditures across revenge conditions for team players and individual player separately. To further examine the dynamics of contest expenditures within the team, we consider individual team members as the unit of observation and introduce lagged variables for the contributions of their own, of their rivals, and of the other three teammates in their teams. The dependent variables also include the dummy variables of the treatments (Unconditional_Revenge_1st, Unconditional_Revenge_2nd, Conditional_Revenge_1st, Conditional_Revenge_Team) and period. The control variables include demographic statistics, such as age, sex and hukou (household registration). Table 1 [1–2] shows the regression results. The coefficients in [1–2] for Unconditional_Revenge_1st, Conditional_Revenge_1st, and Conditional_Revenge_Team are negative and significant. This means that individual team players invested less when faced with unconditional or conditional revenge against the highest investor, or conditional revenge against all investors than when there is no revenge. The coefficients of the period are negative and significant. In addition, the coefficients for their own lagged expenditures are positive and highly significant, which indicates the presence of a considerable degree of path dependency. The coefficients for the rival’s lagged expenditures are much lower than those for the own lagged expenditures, and are statistically significant. We also find that team members appeared to pay attention to the median and the lowest of the other three expenditures but appeared to care little about the highest expenditures.

**Table 1:** Determinants of contest expenditures in the revenge treatments.

	Team X		Team Y		Total	
	[1]	[2]	[3]	[4]	[5]	[6]
Unconditional_Revenge_1st	−1.61***	−1.36**	8.06**	11.57***	3.01	6.67
	(0.60)	(0.60)	(3.15)	(3.29)	(4.05)	(4.23)
Unconditional_Revenge_2nd	−0.68	−0.19	8.31***	10.85***	6.22	9.85**
	(0.60)	(0.62)	(3.14)	(3.46)	(4.04)	(4.44)
Conditional_Revenge_1st	−1.86***	−1.64***	−1.94	−1.08	−7.23*	−8.20*
	(0.60)	(0.62)	(3.21)	(3.39)	(4.13)	(4.35)
Conditional_Revenge_Team	−2.02***	−1.55**	−1.34	−0.14	−7.32*	−6.85
	(0.60)	(0.62)	(3.19)	(3.41)	(4.11)	(4.380)
Period	−0.14***	−0.11***	0.21	0.18	−0.18	−0.20
	(0.04)	(0.04)	(0.19)	(0.19)	(0.24)	(0.240)
L. Own	0.49***	0.47***	0.46***	0.42***		
	(0.02)	(0.02)	(0.03)	(0.03)		
L. Rival	0.06***	0.05***	0.38***			
	(0.005)	(0.01)	(0.04)			
L. Team Y					0.63***	0.57***
					(0.04)	(0.04)
L. Team X					1.01***	
					(0.05)	
L. R1		−0.02		0.37***		0.88***
		(0.02)		(0.09)		(0.12)
L. R2		0.08***		0.33**		0.67***
		(0.03)		(0.15)		(0.20)
L. R3		0.09**		0.51**		1.54***
		(0.04)		(0.24)		(0.30)
L. R4				0.27		1.22***
				(0.25)		(0.32)
Control variables		Yes		Yes		Yes
Constant	5.70***	5.96**	1.580	17.26	17.14***	50.85**
	(0.67)	(2.59)	(3.816)	(16.16)	(4.91)	(20.760)
Obs	3040	3040	760	760	760	760
R^2^	0.37	0.38	0.55	0.57	0.72	0.73

*Notes*: The dependent variables include the expenditures of individual team members on Team X ([1]–[2]), the expenditures of the player on Team Y ([3]–[4]), and the total expenditures of both teams ( [5]- [6]). The independent variables consist of treatment dummies for four revenge treatments, lagged contributions, including those of the individual, and their rivals, as well as period effects. In Columns [1]–[2], L. R1, L. R2, and L. R3 represent the lagged ranked expenditures of teammates, excluding the individuals themselves. In Columns [3]–[6], L. R1, L. R2, L. R3, and L. R4 indicate the ranked expenditures of all teammates on Team X. Control variables include demographic characteristics such as age, sex, and hukou (household registration). The numbers in parentheses are robust standard errors clustered on groups. Significance levels are denoted as *p < 0.10* (*), *p < 0.05* (**), and *p < 0.01* (***).

**Result 1**: Individuals in the payoff subtraction treatments, specifically those facing unconditional subtractions for the highest investor or conditional subtractions applied to either the highest investor or all team members, reduced their competitive expenditures.

Similar analyses are applied for the expenditures of single players on Team Y. We introduce lagged variables for their own contributions and the contributions of their rivals, which are measured via total team contribution and within-group ranked contribution. The dependent variables also include the dummy variables of treatment, period and control variables. Table 1 [3–4] present the regression results. The coefficients for the two conditional revenge treatments are positive and significant. This reveals the surprising finding that unconditional payoff subtractions targeting only one party led to an increase in competitive expenditures by the unaffected party, which can ultimately result in inefficient allocation and waste of competitive resources. The coefficients for own lagged expenditures are also positive and highly significant, which indicates the presence of a considerable degree of path dependency. In addition, single players appear to have paid attention to rivals’ expenditures; more specifically, they paid attention to the top three, but not to the bottom.

**Result 2**: Unconditional payoff subtractions applied to one party led to an increase in competitive expenditures by the party not subjected to the subtractions.

#### 3.2.2 Total expenditure behaviours of both parties.

To determine whether revenge reduced total expenditures, we now look at the behavior of two competitive parties. We compare differences in the total expenditures of both parties across the revenge conditions. Table 1 [5–6] present the regression results. The coefficients of Conditional_Revenge_1st are negative and marginally significant. This finding indicates that conditional revenge against the highest investor slightly reduced competitive expenditures; this concept could mitigate the waste of resources caused by competition. The coefficients of all lagged terms are positive and significant, indicating that total expenditures were significantly affected by the expenditures of the parties in the previous period.

**Result 3**: Conditional payoff subtractions targeting the highest investor slightly reduce total competitive expenditures.

#### 3.2.3 Detailed behavior of individual team members.

To gain more insight into how teams arrive at their expenditure decisions, we now look at the behavior of individual team members. We rank the contest expenditures of the individual team players in each round. Fig 3A shows the variability and distribution of the highest, second-highest, second-lowest, and lowest expenditures across the baseline treatment and four revenge treatments. On the basis of the figure, the average differences between team players were extreme. On average, the highest investor spent almost five times that of the lowest investor in the baseline treatment. The top contributors spent over 30 percent of their endowment across all treatments. Notably, in Unconditional_Revenge_2nd, their expenditures exceeded 60 percent, doubling the amount observed in Conditional_Revenge_1st. And the lowest investor spent approximately 10 percent of their endowment in the baseline treatment on average, and this value decreased to 3.4 percent in Conditional_Revenge_Team. To determine the factors that influence the highest and lowest expenditures within the group, a more in-depth analysis of the data is carried out.

To further examine the dynamics of the highest and lowest contest expenditures within the team, we performed regression analyses on the dummy variables of treatment, period, lagged variables and control variables. The regression results revealed significantly negative effects of Conditional_Revenge_1st on the expenditures of the top investors ([1–2] in S1 Table). A possible explanation is that conditional payoff subtraction against the highest level may have a greater deterrent effect relative to unconditional revenge, which succeeded in reducing the highest level of expenditures within the team. However, revenge had a limited effect on the lowest level of contest expenditures ([3–4] in S1 Table).

### 3.3 Results on the effects of collusion

#### 3.3.1 Time trends and differences between treatments and between conflict parties.

On the basis of fig 4, three observations seem noteworthy. First, there was a downwards trend over time in all the treatments. Second, the overall actual expenditures appear to be higher than the Nash equilibrium prediction. Even towards the end of the experiment, the expenditures in Unconditional_Collusion_3rd, Conditional_Collusion_4th and Conditional_Collusion_Team appear to be higher than in the equilibrium prediction, although the average expenditures seem lower than the equilibrium prediction in the last quarter for Team Y in Unconditional_Collusion_4th. Third, Team X seems to have invested more than Team Y in Conditional_Collusion_4th, and both parties seem to have invested more than they did in the other treatments. Examining the behavior within Team X, we observe significant heterogeneity in competitive expenditures (Fig 3B). Notably, on average, the highest investor in Conditional_Collusion_4th spent nearly twenty-three times more than the lowest investor did, highlighting a substantial disparity in contribution levels. Additionally, the top contributors allocated more than half of their endowments in both the Unconditional_Collusion_4th and Conditional_Collusion_4th treatments. In contrast, the lowest investors contributed the most in Unconditional_Collusion_3rd and the least in Conditional_Collusion_4th, with expenditures ranging from 2 to 15 percent of their endowment. Next, we perform a statistical analysis to determine the underlying regularity.

We next compare differences in expenditures across revenge conditions for team players and the individual player separately. The dependent variables include the dummy variables of the treatments, period, lagged items and controlled variables. To further examine the dynamics of contest expenditures within the team, we consider individual team members as the unit of observation and introduce lagged variables for their own contributions, those of their rivals, and those of the other three teammates on their teams. The control variables include demographic statistics, such as age, sex and hukou (household registration). Table 2 [1–2] shows the regression results. The coefficients of Conditional_Collusion_Team are negative and marginally significant. This means that conditional payoff additions distributed to all team members slightly reduced individual team players’ expenditures. In addition, the coefficients for their own lagged expenditures are positive and highly significant, which indicates the presence of a considerable degree of path dependency. The coefficients for the rival’s lagged expenditures are much lower than the player’s own lagged expenditure coefficients and are statistically significant. The coefficients for the other teammates’ expenditures are positive but marginally significant. The influences of rivals and teammates are relatively minimal.

**Table 2:** Determinants of contest expenditures in Collusion treatments.

	Team X		Team Y		Total	
	(1)	(2)	(3)	(4)	(5)	(6)
Unconditional_Collusion_4th	−0.67	−0.37	−2.95	−1.45	−4.83	−2.35
	(0.58)	(0.59)	(2.98)	(3.13)	(4.00)	(4.22)
Unconditional_Collusion_3rd	0.31	0.00	0.45	−2.20	1.37	−2.73
	(0.58)	(0.60)	(2.97)	(3.12)	(3.97)	(4.21)
Conditional_Collusion_4th	−1.19**	−0.72	1.48	3.24	−2.12	0.83
	(0.58)	(0.59)	(2.99)	(3.31)	(4.00)	(4.46)
Conditional_Collusion_Team	−1.32**	−1.08*	−2.45	−2.13	−6.40	−7.03*
	(0.58)	(0.59)	(3.00)	(3.12)	(4.02)	(4.20)
Period	−0.06*	−0.04	0.04	−0.01	−0.12	−0.19
	(0.03)	(0.03)	(0.18)	(0.17)	(0.24)	(0.23)
L. Own	0.55***	0.54***	0.51***	0.48***		
	(0.02)	(0.02)	(0.03)	(0.03)		
L. Rival	0.05***	0.04***	0.26***			
	(0.01)	(0.01)	(0.03)			
L. Team Y					0.66***	0.63***
					(0.04)	(0.04)
L. Team X					0.92***	
					(0.04)	
L. R1		0.03*		0.11		0.69***
		(0.02)		(0.09)		(0.12)
L. R2		0.05*		0.33**		0.86***
		(0.03)		(0.16)		(0.21)
L. R3		0.07*		0.67***		1.66***
		(0.04)		(0.22)		(0.29)
L. R4				−0.20		0.53*
				(0.22)		(0.30)
Control variables		Yes		Yes		Yes
Constant	4.18***	4.17*	7.53**	7.17	19.19***	14.30
	(0.65)	(2.26)	(3.56)	(14.90)	(4.77)	(20.08)
Obs	3040	3040	760	760	760	760
R^2^	0.40	0.40	0.450	0.52	0.68	0.69

*Notes*: The dependent variables include the expenditures of individual team members on Team X ([1]-[2]), the expenditures of the players on Team Y ([3]-[4]), and the total expenditures of both teams ([5]-[6]). The independent variables consist of treatment dummies for Collusion treatments, lagged contributions, including those of the individual, and their rivals, as well as period effects. In Columns [1]-[2], L. R1, L. R2, and L. R3 represent the lagged ranked expenditures of teammates, excluding the individuals themselves. In Columns [3]-[6], L. R1, L. R2, L. R3, and L. R4 indicate the ranked expenditures of all teammates on Team X. Control variables include demographic characteristics such as age, sex, and hukou (household registration). The numbers in parentheses are robust standard errors clustered on groups. Significance levels are denoted as *p < 0.10* (*), *p < 0.05* (**), and *p < 0.01* (***).

Similar analyses are applied for the expenditures of the single players on Team Y. Table 2 [3–4] present the regression results. The coefficients of the collusion treatments are nonsignificant. The coefficients for the player’s own lagged expenditures are positive and highly significant, which indicates the presence of a considerable degree of path dependency. In addition, the coefficients for rivals’ lagged expenditures are positive and significant, which means that single players appear to have paid attention to the expenditures of the rival team.

#### 3.3.2 Total expenditure behaviours of both parties.

To determine whether collusion reduced the total expenditures, we now look at the behaviour of two competitive parties. We compare differences in the total expenditures of both parties across collusion conditions. Table 2 [5–6] present the regression results. The results suggest that collusion might have had little effect on reducing the total competitive expenditures. In addition, the total expenditures of the two parties were significantly affected by the expenditures of the parties in the previous period.

## 4. Discussion

Although contests between groups rely solely on their members’ voluntary contributions, expenditures made in fighting have been found to be far above equilibrium. The issue of how to reduce overcontribution effectively is still an open question. In this study, we investigated the effects of payoff subtraction (revenge) and the payoff addition (collusion) on group contests. While these mechanisms did not affect the Nash equilibrium prediction, which is based solely on aggregate team contributions, they significantly influenced individual expenditure decisions across different strategic environments.

Our experimental results revealed that the expenditures consistently exceed the predictions of the Nash equilibrium. This is in line with previous findings from contest experiments, which frequently observe excessive effort relative to theoretical predictions [1,19]. Our theoretical model offers a plausible explanation that individuals do not behave as purely self-interested agents who take others’ strategies as fixed, as assumed in classical game theory. Rather, participants may internalize the mutual interdependence of decisions within the group, leading to higher than theoretically expected efforts. In our model, when individuals recognize that their own effort positively correlated with the group’s average performance, which also in turn influences their potential reward, they may have greater incentives to exert effort. These behavioral patterns may be rooted in social preference, such as concerns for fairness, reciprocity, or collective welfare, which was commonly observed in behavioral experiments [13,20,21].

Beyond aggregate patterns, our model also yields predictions at the individual level. Specifically, when other group members contribute substantial effort, the marginal benefit of additional personal effort declines sharply. In such cases, the best response for a rational individual may be to exert no effort. Our experimental data are consistent with this prediction; we observe some participants choose not to contribute in rounds where their teammates’ efforts are relatively high. Such behavior may reflect a sophisticated strategic response to others’ actions. This aligns with findings in contest experiments where equilibrium permits asymmetric strategies and there is substantial variation in individual efforts [1,2].

Although our results indicated that expenditures remained above the equilibrium level, the introduction of conditional payoff subtraction effectively limited the contest expenditures of both parties to no more than 1.4 times the equilibrium level on average. In contrast, in the absence of any subtractions or additions, expenditures exceeded twice the equilibrium level. Notably, under conditional payoff subtraction targeting the top investor, total expenditures were only 58.2 percent of those observed in the non- subtraction scenario. These findings highlight an effective mechanism for reducing excessive competitive expenditures and promoting resource efficiency.

Costly sanctions are commonly used as economic incentives, particularly in social dilemmas. For example, it has been widely suggested that costly punishments can uphold cooperation in public goods games [11,13]. Such punishment towards noncooperation could be a deterrent for defection [22]. In this paper, to achieve the goal of reducing over-expenditure, we set the payoff subtraction to the high investor or the whole group, which greatly affects the response of those might be targeted.

From the perspective of psychology, revenge could alter others’ incentives by imposing a cost following an absence of benefits [23,24]. Because of the presence of unequal power, the advantaged party poses a credible threat to the disadvantaged party. Previous studies have shown that the prospect of suffering revenge can alter people’s behaviours. For example, people refrain from harming the interests of their opponents when they know that the opponents have a strong ability to revenge in bargaining games [25]. In addition, in contests with revenge (i.e., punishment), fear has a disciplining effect on team members, leading to the upwards deviation of their investments [1]. In this study, the fear of experiencing revenge might be an important reason why revenge worked.

Our results also showed that conditional revenge seems more efficient in reducing expenditures. Possible explanations are as follows. On the one hand, revenge is not always necessary to retaliate directly; in some cases, the deterrent effect of revenge can be sufficient to influence others’ behaviours [23]. On the other hand, conditional revenge might create a scenario where individuals are uncertain about whether they might trigger payoff subtraction (revenge). Uncertainty is a central feature in the conceptual model of fear, which leads to anxiety and worry [26], such that individuals feel that they must be constantly aware of their actions to avoid provoking payoff subtraction.

Our findings also show that payoff addition of equal magnitude may be less effective than payoff subtraction, if reducing competitive expenditures is the ultimate goal, whether it is lowering the rival group or lowering both sides. On the one hand, people typically exhibit greater sensitivity to losses than to equivalent gains [27]. This aligns with previous social psychology research, which suggests that the desire to avoid negative feedback is typically stronger than the desire to receive positive feedback [28]. On the other hand, revenge may increase levels of fear and anxiety [26], which leads to lower levels of expenditures in competition. Our findings are similar to those of the cooperation framework, with negative sanctions having a greater on cooperative behaviour than the positive sanctions [12,29].

It seems that equilibrium theory, which based on the assumptions of self-interest and full rationality, may not capture some of the essential elements of human behaviour. In this paper, we find that competitive behaviour is indeed affected by the payoff subtraction mechanism. Our findings contribute to the literature on contests with costly sanctions by showing that structured payoff subtractions (revenge) can serve as a behavioral deterrent, reducing competitive expenditures from the targeted group while altering strategic interactions between teams. Experiments such as ours may provide a solution to the large losses often observed in competitions, such as sociopolitical conflicts and rent seeking.

However, it is important to note that the parties in our experiment hold equal power, and neither revenge nor collusion is optional. Prior studies have shown that asymmetries in group size, wealth, sharing rules, and ability can significantly affect contest behavior [1,30,31]. Future research could compare the frequency of revenge or collusion when both parties have equal or unequal power and the resulting impacts on contest expenditures. Beyond payoff-based interventions, non-monetary interventions such as using social norms to discourage excessive competition, implementing reputational mechanisms that reward restraint, or using framing effects to emphasize long-term cooperation over short-term competition could be effective strategies. Examining how these non-rewarding factors interact with monetary incentives will contribute to a more complete understanding of competitive decision-making and provide new approaches to minimizing resource dissipation. Additionally, we observe zero contributions in our experiment. While our design allows for such behavior implicitly, it does not model exit as an explicit choice. Future research should explore deeper into the motivations and conditions of such decisions, building on recent insights by Adamson & Kimbrough. [32], which highlights the importance of exit as a rational response in strategic environments.

## Supporting information

S1 Table Determinants of the highest and lowest contest expenditures within the team under payoff subtractions. (DOCX)

S1 Data Dataset. (XLSX)

S1 Appendix The extended model. (DOCX)
