# Supplementary material for: Revenge or collusion? An experiment on payoff subtraction and addition in team contests
Source: PLoS One. 2025 Sep 4;20(9):e0331015. doi: 10.1371/journal.pone.0331015 (PMC12410788; doi:10.1371/journal.pone.0331015)
Supplement: S1 Table — (DOCX) [file pone.0331015.s001.docx]

S1 Table. Determinants of the highest and lowest contest expenditures within the team under payoff subtractions.

|  | R1 | | R4 | |
| --- | --- | --- | --- | --- |
| Independent Variables | (1) | (2) | (3) | (4) |
| Unconditional_Revenge_1st | -2.32* | -0.93 | -0.28 | -0.35 |
|  | (1.38) | (1.43) | (0.42) | (0.45) |
| Unconditional_Revenge_2nd | 2.53* | 4.17*** | -0.77* | -1.08** |
|  | (1.38) | (1.51) | (0.43) | (0.47) |
| Conditional_Revenge_1st | -3.76*** | -3.95*** | -0.40 | -0.84* |
|  | (1.41) | (1.48) | (0.43) | (0.46) |
| Conditional_Revenge_Team | -2.07 | -1.54 | -0.74* | -1.12** |
|  | (1.39) | (1.49) | (0.43) | (0.47) |
| Period | -0.15* | -0.11 | -0.07*** | -0.07*** |
|  | (0.08) | (0.08) | (0.03) | (0.03) |
| L. Rival | 0.07*** | 0.04*** | 0.03*** | 0.02*** |
|  | (0.01) | (0.01) | (0.00) | (0.00) |
| L. R1 | 0.45*** | 0.32*** |  | 0.01 |
|  | (0.03) | (0.04) |  | (0.01) |
| L. R2 |  | 0.22*** |  | -0.02 |
|  |  | (0.07) |  | (0.02) |
| L. R3 |  | 0.13 |  | 0.07** |
|  |  | (0.10) |  | (0.03) |
| L. R4 |  | -0.04 | 0.66*** | 0.59*** |
|  |  | (0.11) | (0.03) | (0.03) |
| Age |  | -0.41 |  | -0.17* |
|  |  | (0.32) |  | (0.10) |
| Sex |  | -1.82* |  | 0.47 |
|  |  | (1.05) |  | (0.33) |
| City |  | -0.89 |  | -0.96*** |
|  |  | (1.08) |  | (0.34) |
| Constant | 11.54*** | 20.46*** | 1.04** | 4.71** |
|  | (1.66) | (7.05) | (0.47) | (2.20) |
| Obs | 760 | 760 | 760 | 760 |
| R^2^ | 0.40 | 0.43 | 0.62 | 0.63 |

Notes: The dependent variables include the top expenditure of individual team members in Team X ([1]-[2]), and the bottom expenditure in Team X ([3]-[4]). The independent variables consist of treatment dummies for four revenge treatments, lagged contributions, including those of the individual, and their rivals, as well as and period effects. L. R1, L. R2, L. R3, and L. R4 indicate the ranked expenditures of teammates in Team X. Control variables include demographic characteristics such as age, sex, and hukou (household registration). Numbers in parentheses are robust standard errors clustered on groups. Significance levels are denoted as *p < 0.10* (*), *p < 0.05* (**), and *p < 0.01* (***).
