# Supplementary material for: Revenge or collusion? An experiment on payoff subtraction and addition in team contests
Source: PLoS One. 2025 Sep 4;20(9):e0331015. doi: 10.1371/journal.pone.0331015 (PMC12410788; doi:10.1371/journal.pone.0331015)
Supplement: S1 Appendix — (DOCX) [file pone.0331015.s003.docx]

**Appendix for**

**Revenge or collusion? An experiment on payoff subtraction and addition in team contests**

Jiaxin Yu, Wanjun Zheng

**S1 Appendix. The extended model**

In this part, we allow for general conjectures. Let $\pi_{i}\left( x_{i},X,Y \right)$ denote the payoff of a representative player i on Team X, where $x_{i}$ is the number of contest tokens purchased by player i. E is the initial endowment, and P is the winning prize. X is the sum of contest tokens purchased by player i’s team, and Y is the total number of contest tokens bought by the opponent party. The payoff function of player i on Team X can then be written as follows:

$$\pi_{i}\left( x_{i},X,Y \right)=\frac{E}{n}+\frac{X}{X+Y}\times P-x_{i}.$$

Let $\bar{x}$ be the mean contribution of all other teammates. The payoff function of player i on Team X can then be rewritten as follows:

$$\pi_{i}\text{=}\frac{E}{n}+\frac{x_{i}+(n-1)\bar{x}}{x_{i}+\left( n-1 \right)\bar{x}+Y}\times P-x_{i}$$

Let $\beta_{i}$ be the player i’s conjectural variation with respect to the mean activity of other teammates, i.e., $\beta_{i}=\frac{\partial\bar{x}}{\partial x_{i}}$. The first-order condition can be

$$\frac{d\pi_{i}}{dx_{i}}=\frac{1+\left( n-1 \right)\beta_{i}}{x_{i}+\left( n-1 \right)\bar{x}+Y}\times P-\frac{x_{i}+\left( n-1 \right)\bar{x}}{\left[ x_{i}+\left( n-1 \right)\bar{x}+Y \right]^{2}}\times\left[ 1+\left( n-1 \right)\beta_{i} \right]-1$$

$$=\frac{\left[ 1+(n-1)\beta_{i} \right]YP}{\left[ x_{i}+(n-1)\bar{x}+Y \right]^{2}}-1=0. ①$$

Let $\pi_{j}\left( y_{j},X,Y \right)$ denote the payoff of player $j$ on Team Y. The player’s payoff function in this game can then be written as follows:

$$\pi_{j}\left( y_{j},X,Y \right)=\frac{E}{N_{Y}}+\frac{Y}{x_{i}+(n-1)\bar{x}+Y}\times P-y_{j}.$$

The first-order condition can be

$$\frac{\alpha\pi_{i}}{\alpha Y}=\frac{P}{x_{i}+\left( n-1 \right)\bar{x}+Y}-\frac{YP}{\left[ x_{i}+\left( n-1 \right)\bar{x}+Y \right]^{2}}-1=0. ②$$

Joining equations 1 and 2, we get

$$X^{*}=[1+\left( n-1 \right)\beta_{i}]Y^{*}$$

$${x_{i}}^{*}=\frac{1}{1+\frac{2}{1+\left( n-1 \right)\beta_{i}}+\frac{1}{{(1+\left( n-1 \right)\beta_{i})}^{2}}}P-(n-1)\bar{x}^{*}$$

$$Y^{*}=\frac{1+\left( n-1 \right)\beta_{i}}{{(2+\left( n-1 \right)\beta_{i})}^{2}}P$$

**Prediction 1**: When $\beta_{i}\leq-1/(n-1)$, $X^{*}$ would be zero, which means everyone chooses to exit. When $\beta_{i}>-1/(n-1)$, $X^{*}>0$, which means that at least one member entries.

**Prediction 2**: When $\beta_{i}=0$, we will get Nash equilibrium that $X^{*}=Y^{*}=P/4$. When $\beta_{i}>0$, members in team X invest more than team Y. When $\beta_{i}<0$, members in team X invest less than team Y.

**Prediction 3**: When $\beta_{i}>0$, the larger the $\beta_{i}$, the larger the $x_{i}$.
